# Leptin and Adiponectin Signaling Pathways Are Involved in the Antiobesity Effects of Peanut Skin Extract

**DOI:** 10.1155/2019/2935315

**Published:** 2019-10-14

**Authors:** Lan Xiang, Qiaobei Wu, Lihong Cheng, Kaiyue Sun, Jing Li, Minoru Yoshida, Jianhua Qi

**Affiliations:** ^1^College of Pharmaceutical Sciences, Zhejiang University, Hangzhou 310058, China; ^2^Chemical Genomics Research Group, RIKEN Center for Sustainable Resource Science, Hirosawa, Wako, Saitama 3510198, Japan; ^3^Department of Biotechnology and Collaborative Research Institute for Innovative Microbiology, The University of Tokyo, Yayoi 1-1-1, Bunkyo-ku, Tokyo 113-8657, Japan

## Abstract

Excessive food intake and metabolic disorder promote obesity and diabetes. In China, peanut skin is used as a herbal medicine to treat hemophilia, thrombocytopenic purpura, and hepatic hemorrhage. In the present study, we demonstrated that peanut skin extract (PSE) safely reduced appetite, body weight, fat tissue, plasma TG and TC, and blood glucose level in mice with diet-induced obesity (DIO). Moreover, the leptin/leptin receptor/neuropeptide Y (NPY) and adiponectin signaling pathways involved in the antiobesity effects of PSE are confirmed through leptin and adiponectin overexpression and leptin receptor silencing in mice. PSE consisted of oligosaccharide and polyphenol in a mass ratio of 45 : 55, and both parts were important for the antiobesity function of PSE. Our results suggested that PSE can be developed as functional medical food to treat metabolic disorders and obesity.

## 1. Introduction

As a global epidemic disease, obesity has become one of the greatest public health concerns of the 21st century [1]. In 2014, the World Health Organization estimated that more than 1.9 billion adults are overweight and over 600 million are obese [2]. Obesity strongly increases the risk factors for nonalcoholic fatty liver disease, type 2 diabetes, cardiovascular disease, and cancer [3]. All of these diseases not only severely influence the quality of life but also shorten the lifespan of human [4, 5]. However, most of the commercially available drugs show only marginal effects. The rebound after drug withdrawal and side effects of these drugs are considered the major problems. To overcome some of these limitations, we utilized safe functional foods and dietary regulation for obesity treatment.

Since ancient times, peanuts are popular in China as a longevity nut. Peanut skin is used to treat hemophilia, primary and secondary thrombocytopenic purpura, and hepatic hemorrhage in traditional Chinese medical science. Peanut is rich in phenols and other potentially health-promoting compounds. And phenolic compounds are largely found in peanut skin extract [6]. Phenolic compounds are effective on various diseases, such as diabetes, obesity, cancer, cardiovascular disease, and inflammation [7–11]. Recently, we found that a mixture of PSE and fish oil could improve the memory and learning ability of normal mice [12]. In addition, the novel antiobesity effect of PSE is consistently observed [13–15]. High-fat diet- (HFD-) induced obese mice were used to investigate the antiobesity effects and mechanism of action of PSE under pathological condition.

Adipose tissue is an endocrine organ that produces several adipokines, such as leptin and adiponectin, which are involved in the regulation of energy metabolism, insulin sensitivity, appetite, inflammation, atherosclerosis, and cell proliferation [15, 16]. Leptin is a product of the obese gene, which is mainly produced by adipose tissue and at low levels by the stomach, skeletal muscle, and placenta [17]. Leptin regulates food intake and energy expenditure by targeting the leptin receptor in the arcuate nucleus of the hypothalamus [18]. Leptin inhibits NPY and agouti-related peptide (AgRP), which are orexigenic and increase food intake, and stimulates proopiomelanocortin, which activates anorexigenic factors, such as *α*-melanocyte-stimulating hormone, inhibiting food intake [19–21]. Adiponectin is a protein hormone with 244 amino acids derived from adipose tissue [22]. Adiponectin mainly targets adiponectin receptors (AdipoR1 and AdipoR2) to regulate energy metabolism and exert functions, such as antiatherogenic, anti-inflammatory, antidiabetic, and cardioprotective effect [23–25]. Increased leptin gene expression in adipocytes and increased plasma leptin have been reported in obese individuals [26, 27]. The gene expression of adiponectin and plasma adiponectin in obese patients, pigs, and rodents are also significantly reduced [28, 29]. Therefore, we examined the antiobesity effects of PSE on obese and normal mice and focused on adiponectin and leptin signaling pathways to analyze the mechanism of action. We reported that the adiponectin and leptin signaling pathways were involved in the antiobesity effect of PSE in obese mice.

## 2. Materials and Methods

### 2.1. Preparation of Peanut Skin Extract and Animal Experimental Design

The dried peanut skin (Anjie, Fuzhou, Zhejiang, China) was extracted with a mixture of ethanol and water at ratio of 40 : 60 for three times. The extracts were collected and adsorbed with HP-20 resin. After washing with water, the HP-20 resin was eluted with ethanol solution. The eluents are collected, combined, concentrated, and dried to prepare the PSE. Milli Q water was used as vehicle in this study. The ICR mice at the age of five weeks (*n* = 500) were used as experimental animals (Zhejiang Academy of Medical Sciences, Hangzhou, China). The animals were housed in a clean room at 23 ± 1°C with a 12 : 12 light-dark cycle and fed with a commercial diet [normal diet (ND) or high-fat diet (HFD) with 22.3% crude protein, 19.8% fat, and 44.6% carbohydrate from Silaike Experimental Animal Co. Ltd. Shanghai, China]. All experiments were conducted under the strict adherence to the Guide for the Care and Use of Laboratory Animals of the National Institutes of Health. The protocol was approved by the Committee on the Ethics of Animal Experiments of Zhejiang University (ZJU201604036).

In this experiment, six-week male mice were divided into five groups and each group contained ten animals. The normal control group received vehicle (water) and ND. The HFD control group acquired the vehicle and HFD freely. Three HFD plus PSE groups admitted PSE at 4, 80, and 160 mg/kg body weight per day and HFD freely. The experimental period was for 6 weeks, and the body weight, food intake, and water consumption were recorded every week during this period. At the end of the experiment, blood was collected from the orbit of the mice using the capillary tube. The mice were killed, and the heart, liver, pancreas, spleen, kidney, white adipose tissue, and brain of the mice were weighed and obtained as samples. The organs were stored at -30°C for further analysis. This experiment was repeated three times.

### 2.2. Preparation and Purification of pTarget/ADN, pTarget/LPN, and pTarget vectors

Plasmid DNA-encoding mouse adiponectin (ADN) or leptin (LPN) was constructed, and the preparation and purification of pTarget/ADN, pTarget/LPN, and pTarget vectors were done as described in a previous study [26]. The detailed method was shown in the supporting information.

### 2.3. Overexpression of Adiponectin and Leptin Gene in the Livers of Obese Mice In Vivo

Fifty of the male ICR mice at six weeks old were randomly divided into five groups. The normal control group was fed with a ND, and other groups were given a HFD for one month. The gene transfer of leptin and adiponectin was done as described in a previous study [26]. The detailed method was displayed in the supporting information.

### 2.4. Knockdown of Leptin Receptor in Mice In Vivo

The synthetic siRNA and annealed duplex of 23-nucleotide RNA targeted to a leptin receptor (site 5 or site 6) were purchased from the company Sangon Biotech, Shanghai, China. The siRNA injection was performed with hydrodynamic based gene delivery consistent with other reports [30]. The detailed method was displayed in the supporting information.

### 2.5. Measurement of Fasting Glucose and Biochemical Indexes of Blood

At the end of the experiment, mice were fasted overnight, and the fasting glucose was measured using a glucometer (Andon Health, Tianjin, China)as our previous study [31]. Blood was collected from the mouse orbit using a capillary tube and centrifuged, and the supernatant was obtained. Leptin and adiponectin were measured using mouse leptin and adiponectin ELISA kits (Westang Bio-Tech, Shanghai, China; Cusabio, Wuhan, China) in accordance with the instructions of the manufacturer. The plasma samples (ALT, AST, TG, TC, HDL, and LDL) were measured using assay kits (Roche, Basel, Switzerland).

### 2.6. RT-PCR Analysis

Approximately 100 mg of epididymal fat, liver, and one hypothalamus samples was used to extract RNA. RNA extraction and cDNA synthesis of the white adipose tissue, liver, and hypothalamus were done as described in our previous studies [26]. The detailed method was shown in the supporting information.

### 2.7. Western Blot Analysis

The 200 mg protein sample of each liver, fat tissue, and hippocampus was prepared, and protein concentration was measured as described in a previous study [26]. The detailed method was given in the supporting information.

### 2.8. Assessment of Histological Sections

The samples of the fat tissue and livers were preserved in 10% formalin solution followed by tissue dehydration with alcohol and xylene as our paper [31]. Each sample was then embedded in paraffin wax, sectioned at 5 *μ*m, and mounted on slides prior to staining. Hematoxylin and eosin stains and oil red staining were used. The slides were observed under the light microscope, and the observations were recorded using 20x lenses. The size of fat cells was calculated using Image-Pro plus 6 software (Media Cybernetics, Maryland, USA).

### 2.9. Analysis of Nonoligosaccharide of PSE

The nonoligosaccharide of PSE was directly analyzed by Agilent technologies 6224A accurate mass LC-TOF-MS under the following conditions: Agilent Extend C18 column (3.5 *μ*m, 3.0 mm × 100 mm), detected at 210 nm; *t* = 0 min MeOH/H_2_O/formic acid (5 : 95 : 0.1), *t* = 20 min MeOH/H_2_O/formic acid (45 : 55 : 0.1); and flow rate: 0.45 ml/min. The data were analyzed using Agilent Software (Agilent Mass Hunter Qualitative Analysis B.04.00).

### 2.10. Preparation of Oligosaccharide and Nonoligosaccharide

PSE (1.2 g) was purified by HPLC [Develosil ODS-UG-5 (*ϕ* · 20 × 250 mm), Nomura Chemical, flow rate: 6 ml/min, 0–15 min, 18% aqueous MeOH, isocratic elution; 15–25 min, 18%–100% aqueous MeOH, linear gradient elution; 25–40 min, 100% MeOH, isocratic elution] to obtain oligosaccharide and nonoligosaccharide of 517.5 and 682.5 mg with *t*_*R*_ = 0–15 and 16–40 min, respectively. Furthermore, the carbon and hydrogen spectra of oligosaccharide and nonoligosaccharide fraction were measured by NMR. The antiobesity effects of the three fractions were confirmed *in vivo*, as described in an animal experimental design.

### 2.11. Monosaccharide Composition Analysis of Oligosaccharide Fraction

Approximately 2 mg oligosaccharide fraction was hydrolyzed using 4 ml of 2 M trifluoroacetic acid at 110°C for 2 h. The hydrolysate sample was dried, dissolved in water, and then reduced with NaBH_4_. The samples were acetylated with 0.5 ml pyridine-acetic anhydride at a ratio of 1 : 1 *v*/*v* and 90°C for 1.5 h. In addition, the rhamnose, fucose, arabinose, xylose, mannose, glucose, galactose, D-(+)-chiro-inositol, and myo-inositol of nine monosaccharides were used as standards to quantify the monosaccharide content. The mixture of the standards was reduced and acetylated by using the same method. The resulting alditol acetates were examined by the Agilent 7000C Triple Quadrupole GC/MS System (Agilent Technologies Inc., USA). Samples were analyzed on a HP-5-fused silica column (J&W Scientific Co., USA). The initial oven temperature was held at 60°C for 2 min, raised to 190°C for 1 min at 15°C/min, raised to 240°C for 3 min at 5°C/min, and then increased to 300°C for 5 min at 10°C/min. GC-MS data were analyzed using Agilent Software (Agilent Mass Hunter Qualitative Analysis B.07.00).

### 2.12. Molecular Weight (Mw) Determination of Oligosaccharide Fraction of PSE

The average Mw of the oligosaccharide fraction of PSE was determined by high-performance gel permeation chromatography (HPGPC) with equipment of Waters 515 chromatography, TOSOH BIOSEP G4000SWXL column (7.8 × 300 mm, Tokyo, Japan) and Waters 2410 differential refractive index detector. The 60 *μ*l sample was eluted with 0.2 M Na_2_SO_4_. Different Mw of dextran standards (Sigma-Aldrich Co., St. Louis, MO, USA) (Mw: 4.44, 9.89, 76.9, 188, and 327 kDa) were used for data analysis.

### 2.13. Statistical Analysis

Animal experiments were repeated twice or thrice. The experimental data was presented as the mean value ± SEM, and significant differences between groups were analyzed through one-way ANOVA followed by Tukey's posttest of GraphPad Prism software (GraphPad Prism). ^∗^*p* < 0.05 or ^#^*p* < 0.05 represents a statistically significant difference between the two groups.

## 3. Results

### 3.1. PSE Prevents HFD-Induced Obesity in Mice

**Figure 1:**
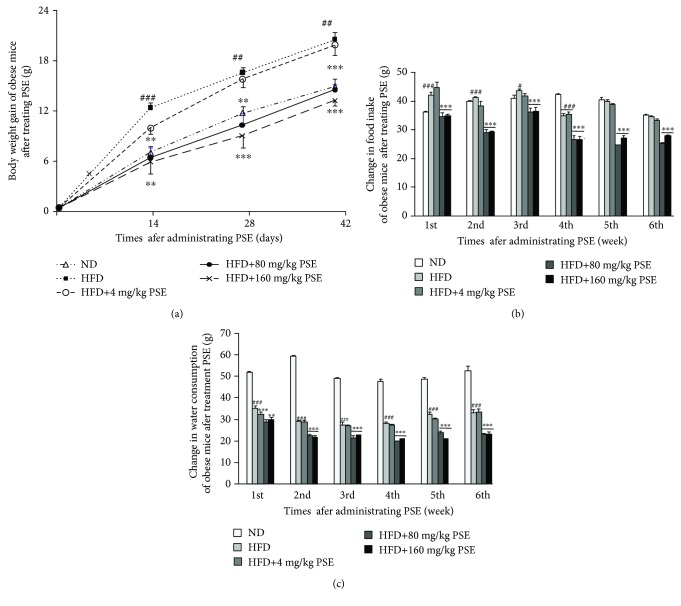
Antiobesity effects of PSE for obese mice. The change of body weight (a), food intake (b), and water consumption (c) of obese mice after administrating PSE at 4, 80, and 160 mg/kg for six weeks. Each point in the figure represents the mean ± SEM at corresponding time points. Animal numbers of each group are ten, and the experiment is repeated three times. #, ##, and ### represent the significant difference comparison with the normal control at *p* < 0.05, *p* < 0.01, and *p* < 0.001. ∗∗ and ∗∗∗ indicate the significant difference compared with the HFD group at *p* < 0.01 and *p* < 0.001.

The changes of body weight, food intake, and water consumption of HFD-induced obese mice are given in Figure 1. The body weight gain and food intake of obese mice are significantly higher than those of normal mice (*p* < 0.001). However, the water consumption of obese mice is evidently lower than that of the normal mice (*p* < 0.001). After administrating PSE with HFD, the body weight gain (Figure 1(a)), food intake (Figure 1(b)), and water consumption (Figure 1(c)) of obese mice are significantly decreased compared with the HFD group at doses of 80 and 160 mg/kg (*p* < 0.01, *p* < 0.001). These results suggest that PSE exhibits antiobesity effects for HFD-induced obese mice.

### 3.2. PSE Reduces Fat Tissue and Regulates Gene Expression and Secretion of Adipocytokine in Obese Mice

**Figure 2:**
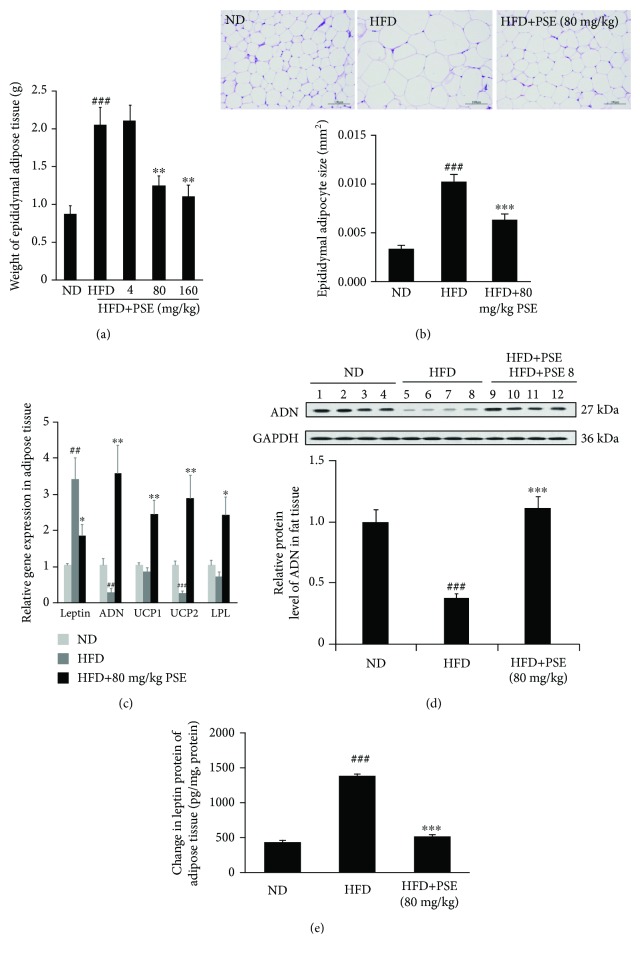
Effects of PSE on adipose tissue of obese mice after PSE treatment. The change of weight of epididymal fat (a); fat cell size (b); gene expressions of leptin, adiponectin, UCP1, UCP2, and LPL (c); adiponectin protein (d); and leptin protein (e) of obese mice after administrating PSE at 80 mg/kg for six weeks. The numbers of samples are seven, and the values represent the mean ± SEM. ## and ### represent the significant difference comparison with the normal control at *p* < 0.01 and *p* < 0.001. ∗, ∗∗, and ∗∗∗ indicate the significant difference comparison with the HFD group at *p* < 0.05, *p* < 0.01, and *p* < 0.001. Scale bar is 100 *μ*M.

The changes of epididymal adipose tissue weight; fat cell size; gene expressions of leptin, adiponectin, UCP1, UCP2, and LPL; leptin and adiponectin protein levels in adipose tissue; and plasma leptin and adiponectin of obese mice are displayed in Figure 2 and Table 1. The epididymal adipose tissue in the HFD group is significantly increased compared with that in the normal control group (Figure 2(a), *p* < 0.001), and the epididymal adipose tissues in the HFD plus PSE groups are dose-dependently reduced at 4, 80, and 160 mg/kg (Figure 2(a), *p* < 0.01). The fat cell size of adipose tissue in the HFD group is significantly increased compared with that in the normal diet (ND) group (Figure 2(b), *p* < 0.001), and the fat cell size of adipose tissue after PSE treatment is significantly smaller than that of the HFD group (Figure 2(b), *p* < 0.001). The gene expressions of adiponectin and UCP2 in the HFD group are significantly decreased compared with the normal control group (Figure 2(c); *p* < 0.01, *p* < 0.001). However, these gene expressions are significantly increased after PSE treatment (Figure 2(c), *p* < 0.01). The gene expression of leptin in the HFD group is significantly increased but is reduced by PSE (Figure 2(c); *p* < 0.05, *p* < 0.01). Furthermore, the adiponectin in adipose tissue and plasma adiponectin in the HFD plus PSE group are also higher than those in the HFD control group (Figure 2(d), *p* < 0.001; Table 1, *p* < 0.05). By contrast, the leptin of adipose tissue and plasma leptin in the HFD plus PSE group is lower than that of the HFD control group (Figure 2(e), *p* < 0.001; Table 1, *p* < 0.001). These results suggest that PSE stimulates adiponectin secretion of adipose tissue, increases sensitivity of leptin, and promotes lipid decomposition metabolism of obese mice.

### 3.3. PSE Eliminates Fatty Liver and Regulates Gene Expressions in the Liver of Obese Mice

**Figure 3:**
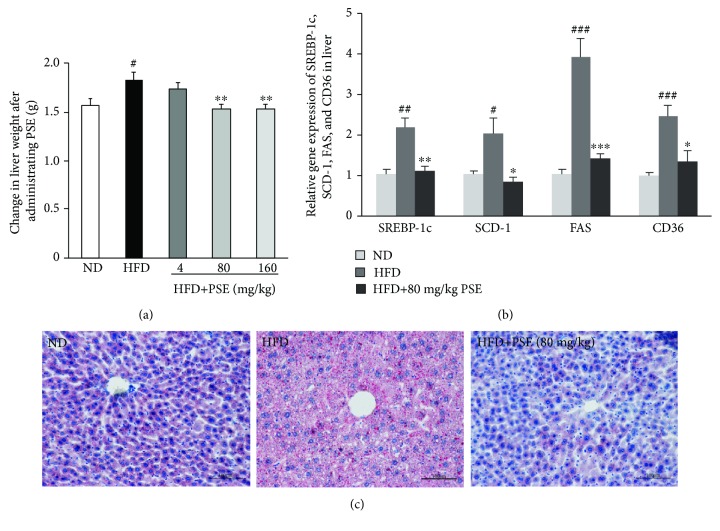
Effects of PSE on livers of obese mice. The changes of liver weight (a); gene expressions of SREBP-1c, SCD-1, FAS, and CD36 (b); and cell morphology of the liver (c) of obese mice after administrating PSE. The numbers of samples are seven, and the values represent the mean ± SEM. ^#, ##, and ###^ represent the significant difference comparison with the normal control at *p* < 0.05, *p* < 0.01, and *p* < 0.001. ∗, ∗∗, and ∗∗∗ indicate the significant difference comparison with the HFD group at *p* < 0.05, *p* < 0.01, and *p* < 0.001. Scale bar is 100 *μ*M.

The changes of liver weight, morphological changes of liver cells, and gene expression-related adipogenesis in the liver of obese mice are shown in Figure 3. The significant increase of liver weight in the HFD control group is observed (Figure 3(a), *p* < 0.05). The liver weight of obese mice is reduced by PSE at doses of 80 and 160 mg/kg (Figure 3(a); *p* < 0.01, *p* < 0.01). Gene expression-related adipogenesis, such as sterol-regulatory element-binding protein-1c (SREBP-1c), stearoyl-CoA desaturase-1 (SCD-1), fatty acid synthase (FAS), and cluster of differentiation 36 (CD36) in the liver of obese mice, is higher than those of normal the control group (Figure 3(b); *p* < 0.05, *p* < 0.01, and *p* < 0.001). Simultaneously, the mRNA abundance of these genes in obese mice is significantly decreased by administrating PSE compared with the HFD control group (Figure 3(b); *p* < 0.05, *p* < 0.01, and *p* < 0.001). The marked steatosis and fatty infiltration of liver cells are found in the liver of the HFD group, and these changes of the liver cells significantly disappear after administrating PSE at a dose of 80 mg/kg (Figure 3(c)). These results indicate that PSE can rescue a fatty liver induced by HFD and reduces liver weight via inhibition of liver adipogenesis in mice.

### 3.4. PSE Regulates the Gene Expressions in the Hypothalamus of Obese Mice

**Figure 4:**
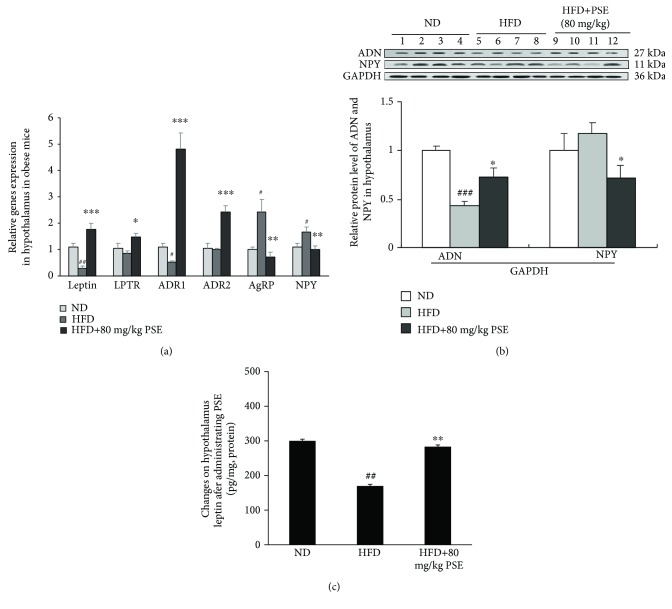
Effects of PSE on the hypothalamus of obese mice. Gene expression of leptin, leptin receptor, ADR1, ADR2, AgRP, and NPY in obese mice after PSE treatment (a). The changes of adiponectin and NPY (b) and leptin proteins (c) in the hypothalamus of obese mice after PSE treatment. Sample numbers are seven or eight, and the values represent the mean ± SEM. #, ##, and ### represent the significant difference comparison with the normal control at *p* < 0.05, *p* < 0.01, and *p* < 0.001. ∗, ∗∗, and ∗∗∗ indicate the significant difference comparison with the HFD group at *p* < 0.05, *p* < 0.01, and *p* < 0.001.

The changes of gene expression-related food intake and energy metabolism in the hypothalamus of obese mice are described in Figure 4. The increase of AgRP and NPY and reduction of leptin and ADR1 gene expressions are observed in the HFD control group (Figure 4(a); *p* < 0.05, *p* < 0.01). The mRNA abundance of leptin, leptin receptor, and ADR1 and ADR2 in the HFD plus PSE group is significantly increased compared with that in the HFD control group (Figure 4(a); *p* < 0.05, *p* < 0.01, and *p* < 0.001). Only ADR1 and ADR2 gene expressions of the hypothalamus in the PSE-treated group are significantly increased (Figure 4(a); *p* < 0.05, *p* < 0.01). Furthermore, the protein levels of adiponectin and leptin are evidently increased in the HFD plus PSE group (Figures 4(b) and 4(c)). Meanwhile, the protein level of NPY is significantly decreased in the HFD plus PSE group (Figure 4(b)). These results suggest that the leptin and adiponectin signaling pathways influence the antiobesity effects of PSE in obese mice.

### 3.5. PSE Changes Blood Biochemical Parameters of Obese Mice

The changes in blood biochemical parameters of obese mice after administrating PSE are revealed in Table 1. The plasma TG, TC, HDL, LDL, and GLU in the HFD group were significantly increased compared with those in the ND group (*p* < 0.05, *p* < 0.001). After administrating PSE for six weeks, the plasma TG, TC, and GLU in the HFD plus 80 and 160 mg/kg PSE groups are significantly decreased (*p* < 0.05, *p* < 0.01, and *p* < 0.001). These results suggest that PSE only intervenes with the obesity induced by HFD.

### 3.6. Leptin and Adiponectin Gene Transfers Reverse HFD-Induced Obesity in Mice

**Figure 5:**
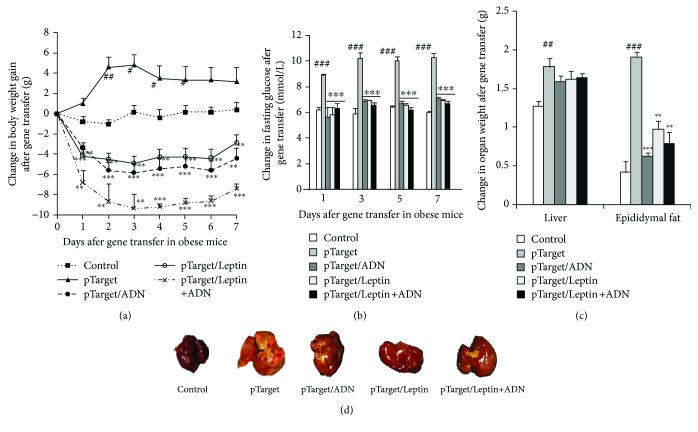
Leptin and adiponectin gene transfer produces the antiobesity effects in obese mice. The changes of body weight (a), fasting plasma glucose (b), weights of epididymal fat tissue and liver (c), and morphological changes of the liver (d) of obese mice after gene transfer and PSE treatment. Each point in the figure represents the mean ± SEM at corresponding time points. Animal numbers of each group are seven. #, ##, and ### represent the significant difference comparison with the normal control at *p* < 0.05, *p* < 0.01, and *p* < 0.001. ∗, ∗∗, and ∗∗∗ indicate the significant difference comparison with the HFD group at *p* < 0.05, *p* < 0.01, and *p* < 0.001.

To indicate whether the leptin and adiponectin signaling pathways influence the antiobesity effects of PSE, we used HFD to induce obesity in obese mice for one month and transferred leptin, adiponectin, and leptin plus adiponectin gene in obese mice *in vivo*. The changes in body weight gain, blood glucose, fat tissue and liver weights, the liver changes, food intake, water consumption, and adiponectin and leptin gene expression in the liver of obese mice after gene transfer are given in Figures 5(a)–5(d) and Supplementary [Supplementary-material supplementary-material-1]. The body weight gains of obese mice after leptin, adiponectin, and leptin plus adiponectin gene transfer for six days are reduced by 232.5%, 268.75%, and 362.1% compared with the pTarget vector-treated group (Figure 5(a), *p* < 0.001). The fasting glucose in the end of experiment (Figure 5(b)) and fat tissue and liver weights of the obese mice (Figures 5(c) and 5(d)) are significantly decreased after leptin, adiponectin, and leptin plus leptin gene transfer which are the same to that of the obese mice after PSE treatment (*p* < 0.05, *p* < 0.01, and *p* < 0.001). The food intake and water consumption of obese mice after gene transfer are significantly lowered by leptin and adiponectin gene transfers (Supplementary [Supplementary-material supplementary-material-1]; *p* < 0.01, *p* < 0.001). The significant increases of adiponectin and leptin gene expression in pTarget/ADN and pTarget/Leptin+ADN were observed at the end of experiment (Supplementary [Supplementary-material supplementary-material-1]; *p* < 0.01, *p* < 0.001). These results suggest that the leptin and adiponectin signaling pathways are involved in the antiobesity effect of PSE.

### 3.7. Leptin Receptor siRNA Diminishes the Antiobesity Effects of PSE in Mice In Vivo

**Figure 6:**
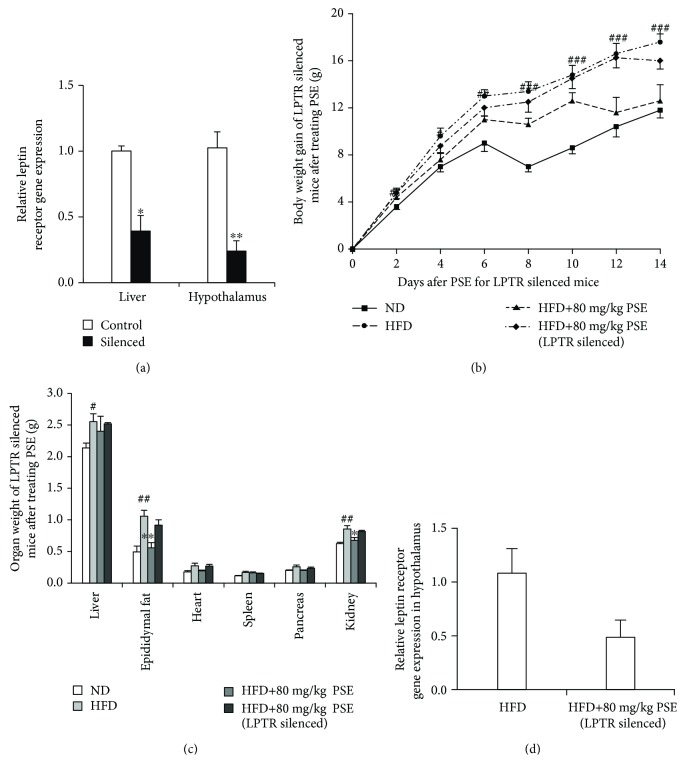
Silence of leptin receptor with siRNA restrains the antiobesity effects of PSE in obese mice. The changes of leptin receptor gene expression in the liver and hypothalamus of normal mice (a) after injection of leptin receptor siRNA, body weight gain (b), organ weight (c), and gene expression of leptin receptor (d) in the hypothalamus of obese mice after injection of siRNA and administration of PSE. Each point in the figure represents the mean ± SEM at corresponding time points. Animal numbers of each group are five. #, ##, and ### represent the significant difference comparison with the normal control at *p* < 0.05, *p* < 0.01, and *p* < 0.001. ∗ and ∗∗ indicate the significant difference comparison with the HFD group at *p* < 0.05 and *p* < 0.01.

To indicate whether the leptin receptor siRNA can knock down leptin receptor gene expression in the liver and hypothalamus, we injected siRNA of leptin receptor with hydrodynamics-based gene delivery in the liver and hypothalamus. The gene expression of leptin receptor in the liver and hypothalamus after injection of leptin receptor siRNA is given in Figure 6(a) and Supplementary [Supplementary-material supplementary-material-1]. The gene expression is significantly decreased compared with the control group (*p* < 0.05 and *p* < 0.01). We used this siRNA of leptin receptor to silence leptin receptor in mice *in vivo* and fed HFD and 80 mg/kg PSE. The changes in body weight, organ weights, food intake, and water consumption of obese mice after treatment of siRNA and PSE are shown in Figures 6(b) and 6(c) and Supplementary [Supplementary-material supplementary-material-1], respectively. The antiobesity effects of PSE for body gain, organ weight, and food intake and water consumption of obese mice are eliminated after treatment of leptin receptor siRNA. The gene expression of leptin receptor in the hypothalamus after treatment of siRNA and PSE for 2 weeks is given in Figure 6(d). The reduction trend of leptin receptor gene expression of the hypothalamus in the PSE plus siRNA-treated group after siRNA treatment for two weeks is existence compared with the HFD-treated group. These results suggest that the leptin receptor siRNA knocks down the leptin receptor gene expression not only in the liver but also in the hypothalamus, and the leptin signaling pathway plays important roles in the antiobesity effects of PSE.

### 3.8. The Components of PSE and Antiobesity Effects of Oligosaccharide and Nonoligosaccharide Fractions of PSE for Obese Mice

**Figure 7:**
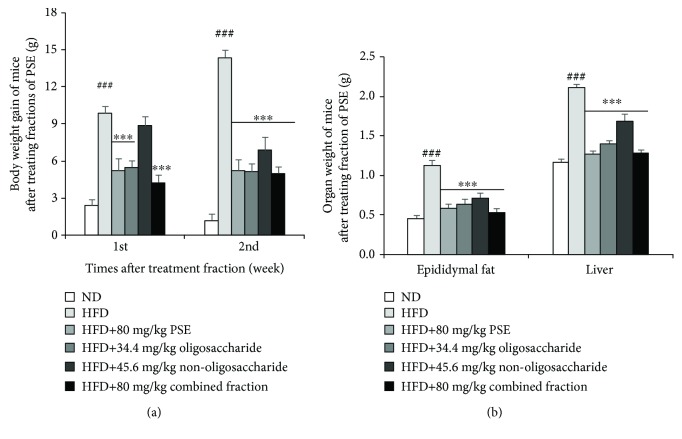
Antiobesity effects of oligosaccharide, nonoligosaccharide, and combined fractions on obese mice. Changes of body weight (a), weights of the epididymal fat tissue and liver (b) after administrating fractions of PSE. Each point in the figure represents the mean ± SEM at corresponding time points. Animal numbers of each group are eight. ### represents the significant difference comparison with the normal control at *p* < 0.001. ∗∗∗ indicates the significant difference comparison with the HFD group at *p* < 0.001.

To clarify the chemical components of PSE, we used HPLC to divide PSE into oligosaccharide and nonoligosaccharide fractions. These fractions' ^1^H-NMR and ^13^C-NMR spectra were analyzed. By comparing with the typical peaks of ^13^C-NMR of oligosaccharide and nonoligosaccharide fractions, we found that the peaks in oligosaccharide (*δ*_C_ = 103.6, 92.1) were not detected in the nonoligosaccharide fraction. Therefore, these two parts were separated completely (Supplementary Figures [Supplementary-material supplementary-material-1]). Subsequently, we analyzed the oligosaccharide fraction and found that it is composed of arabinose, xylose, D-(+)-chiro-inositol, myo-inositol, mannose, glucose, and galactose in a molar ratio of 3 : 6.9 : 1 : 1.5 : 3.2 : 21.5 : 3.8. Glucose and xylose are the main components of the oligosaccharide fraction of PSE (Supplementary [Supplementary-material supplementary-material-1]). The average Mw of the oligosaccharide fraction of PSE is determined by HPGPC. Based on the calibration curve established by dextran standards, the average Mw of the oligosaccharide fraction of PSE is 972 Da (Supplementary [Supplementary-material supplementary-material-1]). In addition, we used high-resolution ESI-MS measurement to analyze nonoligosaccharide fractions. The results in Supplementary [Supplementary-material supplementary-material-1] and Supplementary [Supplementary-material supplementary-material-1] indicate that it mainly consists of the dimer, trimer, and tetramer of A- and B-type procyanidin and other compounds. To determine the most antiobesity active fraction of PSE, we used the three fractions to elucidate the antiobesity effects in obese mice. The body weight gain and the weight of the epididymal fat tissue and liver are significantly decreased after PSE treatment with three fractions (Figures 7(a) and 7(b)). The antiobesity effects of oligosaccharide of PSE are stronger than that of the nonoligosaccharide fraction, and the antiobesity effects of the combined fraction of oligosaccharide and nonoligosaccharide are the best among the three fractions. The significant reduction of food intake and water consumption of mice is observed in the fraction treated-groups (Supplementary [Supplementary-material supplementary-material-1]). These results suggest that both oligosaccharide and nonoligosaccharide fractions of PSE play important roles in the antiobesity effects of PSE.

## 4. Discussion

To get the optimum dose relationship of PSE, we used normal and obese mice to examine several doses of PSE such as 4, 20, 40, 80, and 160 mg/kg. The results in Figure 1, Supplementary [Supplementary-material supplementary-material-1], and Supplementary [Supplementary-material supplementary-material-1] indicated that PSE displayed better dose-dependent manner at 4, 80, and 160 mg/kg. Furthermore, we used these doses to test the antiobesity effects of PSE with obese mice. The significant reductions in body weight and adipose tissue weight after administrating PSE, as shown in Figures 1(a)–2(a), are consistent with other reports [14–16]. We found the significant decrease of food intake and water consumption in the PSE-treated groups, as shown in Figures 1(b) and 1(c). These results suggest that PSE can restrain the increase in the body weight of obese mice induced by HFD not only by decreasing fat deposition but also by lowering appetite and water intake. PSE is different from peanut sprout extract and resveratrol, which did not affect food intake [13, 14]. PSE possesses other bioactive compounds that can lower the appetite of mice that must be indicated in the future.

The main features of obesity are increasing in fat cell numbers and size. Obesity has been prevented by reducing the differentiation of fibroblastic preadipocytes to mature adipocytes and inhibiting lipogenesis [32]. In the present study, HFD significantly increases the weight and size of fat cells of adipose tissue, whereas both of them are significantly decreased after administrating PSE (Figures 2(a) and 2(b)). These results suggest that PSE essentially prevents fat synthesis and accelerates the breakdown of fat to produce antiobesity effects.

The liver is another important organ that prevents obesity. Obesity is often accompanied by hepatic lipid accumulation and development of a fatty liver [33]. The reduction of liver weight and removal of the fat in liver cells after the administration of PSE (Figures 3(a) and 3(c)) indicate that PSE may prevent hepatic lipid accumulation. SREBP-1c is a transcription factor that regulates the expression of downstream target genes, such as FAS and SCD-1, which are involved in glucose utilization and fatty acid synthesis [33]. FAS and SCD-1 are the central lipogenic proteins, along with CD36, are integral membrane protein importing fatty acids inside the cells, and contribute to energy storage by increasing fatty acid uptake in the liver. The significant increase and reduction of these gene expressions of the liver and morphological changes of the liver cells in the HFD group and HFD plus PSE-treated group (Figure 3) indicate that the lipogenesis-inhibiting effects of PSE may be mediated by repressing fatty acid uptake via inhibition of SREBP-1c, FAS, SCD-1, and CD36, and PSE significantly affects nonalcoholic fatty liver disease.

We found that the plasma leptin in obese mice is very high (Table 1), but the leptin that entered into the hypothalamus is lower (Figure 4(c)). The plasma leptin concentration is lowered after the reduction of body weight, and the leptin in the hypothalamus is elevated (Table 1 and Figure 4(c)). This phenomenon has demonstrated that the permeability of the blood brain barrier (BBB) plays an important role in the entry of leptin into the hypothalamus and control of appetite. The increase of BBB permeability and specific transport of leptin may improve the antiobesity efficiency of leptin under the obese condition. The chylous blood of obese mice is observed after feeding HFD to mice for a long time; this symptom of the obese mice is eliminated after administrating PSE (data not shown). High blood lipids may reduce BBB permeability and prevent plasma leptin from entering the hypothalamus. PSE can clear blood fat to improve the permeability of BBB and increase the leptin signal transduction in the hypothalamus. We needed to indicate this feature in the future study.

During the analysis of the action mechanism, the reduction of adiponectin gene expression, adiponectin protein level in the adipose tissue and hypothalamus, and plasma adiponectin in HFD group, as well as the increase of these factors in the HFD plus PSE group (Figures 2(b) and 2(c) and Table 1), demonstrate that PSE is similar to resveratrol and peanut sprout extract in producing antiobesity effects via regulation of adiponectin signaling pathways. In addition, the leptin signaling pathways of mice in the HFD and HFD plus PSE groups are also evidently changed. The significant increase of leptin gene expression, leptin protein level in adipose tissue and plasma leptin, and reduction of leptin in the hypothalamus of mice in the HFD group (Figures 2(b) and 4(c)) are consistent with other reports [26, 27]. The results displayed in Figures 2(c), 2(e), Table 1 and Figure 4(c) suggest that PSE can improve the leptin resistance of obese mice induced by HFD and leptin/leptin receptor/AgRP/NPY and adiponectin/ADR signaling pathways involved in the antiobesity effects of PSE for obese mice. We used the overexpression of leptin and adiponectin and knockdown of leptin receptors to confirm the important role of leptin and adiponectin signaling pathways (Figures 5 and 6, Supplementary [Supplementary-material supplementary-material-1] and [Supplementary-material supplementary-material-1]). We found that adiponectin gene transfer in mice *in vivo* under normal and pathological conditions can produce opposite function. The food intake and body weight gain of normal mice are significantly increased after adiponectin gene transfer [34]. These factors evidently decrease in obese mice after adiponectin gene transfer as shown in Figure 5.

We also investigated the safety, as well as hypolipidemic and hypoglycemic, effects of PSE, in obese mice. The changes of ALT, AST, TG, TC, and GLU shown in Table 1 confirm that PSE is a highly safe bioactive fraction and exhibits novel hypolipidemic and hypoglycemic effects for obese mice.

PSE mainly contains the dimer, trimer, and tetramer of A- and B-type procyanidin and high content of oligosaccharide (Supplementary [Supplementary-material supplementary-material-1] and Supplementary [Supplementary-material supplementary-material-1] and [Supplementary-material supplementary-material-1]). Given that our sample was treated by a specific column, no resveratrol is observed, unlike the reported peanut skin extracts, which contain high resveratrol [35]. Furthermore, we subdivided PSE components into oligosaccharide and nonoligosaccharide. We measured the antiobesity effects of these fractions with HFD-induced obese mice *in vivo*. The results in Figure 7 and Supplementary [Supplementary-material supplementary-material-1] elucidate that the oligosaccharide and nonoligosaccharide fractions in PSE exhibit similar important roles for the antiobesity effects of PSE. Interestingly, the effects of the nonoligosaccharide fraction of PSE were delayed compared with those of the oligosaccharide fraction. Therefore, we considered whether both oligosaccharide and nonoligosaccharide fractions show those effects by regulating the same signaling pathways. The deep research for this problem should be done in the future.

**Figure 8:**
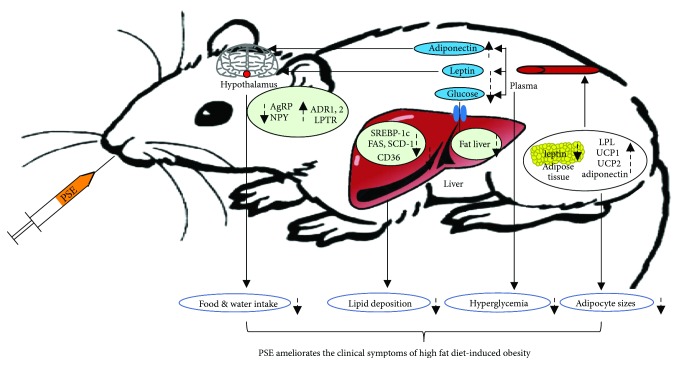
Proposed antiobesity mechanism of PSE for obese mice. PSE produces antiobesity effects in obese mice via regulation of the leptin and adiponectin signaling pathways.

In summary, we found that PSE exhibits antiobesity effects for obese mice, and the leptin and adiponectin signaling pathways play important roles (Figure 8). PSE may be developed as supplementary functional medical food to treat hyperlipidemia and obesity.

## Figures and Tables

**Table 1:** Effects of PSE on blood biochemical parameters of obese mice.

Groups	ALT (U/l)	AST (U/l)	TG (mmol/l)	TC (mmol/l)	HDL (mmol/l)	LDL (mmol/l)	GLU (mmol/l)	LEP (pg/ml)	ADP (*μ*g/ml)
ND	34.00 ± 2.58	119.57 ± 5.26	1.71 ± 0.08	2.97 ± 0.25	2.91 ± 0.23	0.41 ± 0.05	5.74 ± 0.20	141.43 ± 3.39	15.20 ± 0.62
HFD	30.57 ± 2.18	116.00 ± 14.56	2.05± 0.12^#^	4.85±0.27^###^	4.51±0.23^###^	0.90±0.07^###^	8.15±0.30^###^	1526.54±6.88^###^	10.67±0.37^###^
HFD+4 mg/kg PSE	33.71 ± 4.64	110.86 ± 5.46	1.89 ± 0.02	4.89 ± 0.46	4.58 ± 0.38	0.99 ± 0.20	9.13 ± 0.37	—	—
HFD+80 mg/kg PSE	25.86 ± 0.34	110.71 ± 8.57	1.66 ± 0.11^∗^	4.07 ± 0.17^∗^	3.92 ± 0.23	0.76 ± 0.10	6.23±0.28^∗∗∗^	449.34±7.91^∗∗∗^	15.45±0.45^∗∗∗^
HFD+160 mg/kg PSE	29.43 ± 1.04	115.57 ± 5.57	1.52±0.10^∗∗^	4.17 ± 0.14^∗^	4.40 ± 0.19	0.73 ± 0.13	6.56±0.29^∗∗^	—	—

ND: normal dietary; HFD: high-fat diet; ALT: alanine aminotransferase; AST: aspartate aminotransferase; TG: triglyceride; TC: total cholesterol; HDL: high-density lipoprotein; LDL: low-density lipoprotein; GLU: glucose; LEP: leptin; ADP: adiponectin. The sample number is 7. # and ### represent the difference between the ND group and HFD group. ∗, ∗∗, and ∗∗∗ represent the difference between the HFD group and PSE treatment groups.

## Data Availability

The data used to support the findings of this study are included within the article.

## Abbreviations

ADN: Adiponectin
CD36: Cluster of differentiation 36
FAS: Fatty acid synthase
HFD: High-fat diet
ND: Normal diet
LPN: Leptin
PSE: Peanut skin extract
SCD-1: Stearoyl-CoA desaturase-1
SREBP-1c: Sterol-regulatory element-binding protein-1c.

## Supplementary Materials

Supplementary Materials The detailed method of preparation and purification of pTarget/ADN, pTarget/LPN, and pTarget vectors; overexpression of adiponectin and leptin gene in the livers of obese mice in vivo; knockdown of leptin receptor in mice in vivo; RT-PCR analysis; and Western blot analysis.
