# Supplementary material for: Leptin and Adiponectin Signaling Pathways Are Involved in the Antiobesity Effects of Peanut Skin Extract
Source: Oxid Med Cell Longev. 2019 Oct 14;2019:2935315. doi: 10.1155/2019/2935315 (PMC6815585; doi:10.1155/2019/2935315)
Supplement: Supplementary Materials — The detailed method of preparation and purification of pTarget/ADN, pTarget/LPN, and pTarget vectors; overexpression of adiponectin and leptin gene in the livers of obese mice in vivo; knockdown of leptin receptor in mice in vivo; RT-PCR analysis; and Western blot analysis. [file 2935315.f1.docx]

**Supplementary information**

Leptin and adiponectin signaling pathways are involved in the anti-obesity effects of peanut skin extract

Lan Xiang^1^*, Qiaobei Wu^1^, Lihong Cheng^1^, Kaiyue Sun^1^, Jing Li^1^, Minoru Yoshida^2,3^, Jianhua Qi^1^*

^1^ College of Pharmaceutical Sciences, Zhejiang University, Hangzhou 310058, China

^2^ Chemical Genomics research group, RIKEN Center for Sustainable Resource Science, Hirosawa, Wako, Saitama 3510198, Japan

^3^ Department of Biotechnology and Collaborative Research Institute for Innovative Microbiology, The University of Tokyo, Yayoi 1-1-1, Bunkyo-ku, Tokyo 113-8657, Japan

*Corresponding author

Tel./Fax: +86-571-88208627

E-mail address: lxiang@zju.edu.cn (L. Xiang); qijianhua@zju.edu.cn (J.H. Qi).

**Materials and Methods**

***1.1. Preparation and purification of pTarget/ADN, pTarget/LPN, and pTarget vectors.*** Plasmid DNA-encoding mouse adiponectin or leptin was constructed as described in previous study [27]. In brief, total RNA of fat tissue was extracted using an RNA extraction kit (Beijing Cowin Biotechnology, Beijing, China) to prepare the cDNA clone of adiponectin and leptin. Reverse transcription polymerase chain reaction (RT‐PCR) was used to retrieve the full-length mouse adiponectin and leptin cDNA. The amplified products were analyzed by electrophoresis and gel-purified using a DNA purification kit (Beijing Cowin Biotechnology, Beijing, China). The cDNA of adiponectin or leptin was inserted into the pTarget vector through overhang with pTarget™ mammalian expression vector system. The reaction regents were transformed into TOP10 competent cells. The cells were smeared on ampicillin plates and incubated at 37 °C overnight. The clones formed on the agar plates were individually amplified. The plasmid DNA was extracted using DNA purification Kit (Beijiang Cown Biotechnology, Beijing, China) and cut with EcoR I (Takara, Otsu, Japan). The size of the inserted fragment and pTarget vector was checked by 1% gel. Individual adiponectin and leptin clones were subsequently obtained from each pTarget/adiponectin (pTarget/ADN) or pTarget/leptin (pTarget/LPN) and were then sequenced to confirm the fidelity of mouse adiponectin and leptin. Large amplification of the pTarget, pTarget/ADN, and pTarget/LPN were prepared using the Endo Free Plasmid Maxi Kit (Qiagen, Shanghai, China) to remove bacterial endotoxins. The plasmid DNA concentration was measured with Nucleic Acid Protein Analyzer (Eppendorf, Hamburg, Germany).

***1.2. Overexpression of adiponectin and leptin gene in the livers of obese mice in vivo.*** Fifty of the male ICR mice at six weeks old were randomly divided into five groups. Normal control group was fed with ND, and other groups were given HFD for one month. As described in our previous study, 15 μg of DNA (pTarget empty vector, pTarget/ADN, pTarget/LPN, and pTarget/ADN+pTarget/LPN) was added in **physiological saline** solution (0.1 ml/g body weight) and smoothly injected into the tail vein of HFD-induced obese mice. This process was finished within 10 s. **Physiological saline** solution (0.1 ml/g body weight) was also injected into the tail vein of the mice in the normal control group. During the subsequent week, the food intake, changes in body weight, and fasting glucose concentration of mice were recorded. At designated time points, blood was collected for plasma leptin, adiponectin, and other biochemical indicator analyses. At the end of the experiment, treated mice were sacrificed by neck dislocation, and the liver, heart, kidney, epididymal white fat, pancreas, hypothalamus were removed quickly, weighed, and stored at -30 °C for further analysis.

***1.3. Knockdown of leptin receptor in mice in vivo.*** The synthetic siRNA and annealed duplex of 23-nucleotide RNA targeted to leptin receptor (site 5 or site 6) were purchased from the company of Sangon Tech, Shanghai, China. The sequences of synthetic RNA were as follows: sense 5’ -r GCU UCA GUA GUG AAG GCU UTT -3’ , antisense 5’ -r AAG CCU UCA CUA CUG AAG CTT-3’ for site 886; sense 5’ -r GGA GUU CAC CUC AAG UCU UTT-3’; antisense 5’ -r AAG ACU UGA GGU GAA CUC CTT-3’ for 1511; sense 5’ -r GCU UAG AAU UCC CUC GAA UTT-3’, and antisense, 5’ -r AUU CGA GGG AAU UCU AAG CTT-3’for site 2901. To indicate which siRNA can silence leptin receptor gene expression, we injected all three siRNA in the mice by hydrodynamic based-gene delivery, respectively. All of the three siRNA can inhibit leptin receptor gene expression in the liver (Supplementary Figure 3). We selected siRNA for site 1511 to perform the experiment. Five-week-old male ICR mice (approximately 22 g body weight) were purchased from Zhejiang Academy of Medical Sciences, Hangzhou, China. siRNA injection was performed with hydrodynamic based-gene delivery consistent with other reports^34^. Mice received one intravenous injection of 2.2 ml saline containing synthetic siRNA (16 μg) within 5 s on day 0. Subsequently, these mice were fed with HFD and PSE for 2 weeks. The food intake, changes in body weight, and water consumption of the mice were recorded.

***1.4. RT-PCR analysis.*** Approximately 100 mg of epididymal fat, liver, and one hypothalamus samples were used to extract RNA. RNA extraction and cDNA synthesis of white adipose tissue, liver, and hypothalamus were done as described in our previous studies^27^. The transcription levels of mRNA were quantified with CFX96-Touch (Bio-Rad, Hercules, USA) and SYBR Premix EX Taq^TM^ (Takara, Otsu, Japan). The primers (Sangon Tech, Shanghai, China) in this study are displayed in Supplementary Table 1. We amplified cDNA using the following conditions: 95 °C for 2 min, followed by 40 cycles for 15 s at 95 °C, and 35 s at 60 °C. All results were standardized to *18S* RNA gene expression, and relative mRNA transcript levels were computed by the ΔΔCt formula.

***1.5. Western blot analysis.*** The 200 mg protein sample of each liver, fat tissue, and hippocampus was prepared, and protein concentration was measured as described in a previous study^27^. Fifty microgram protein of each sample was loaded onto the 10% or 13% SDS-PAGE gel and run at 120 V for 60 min. The protein on the gel was transferred to polyvinylidene difluoride membranes. The membrane was blocked with 5% non-fat milk in TBPS for 60 min, incubated first with antibodies of adiponectin, NPY (Cell Signaling Technology, MA, USA), and GAPDH (Beijing ComWin Biotechnology, Beijing, China) in 2% non-fat milk overnight at 4 °C. Afterward, the membrane was incubated with a secondary antibody (Beijing ComWin Biotechnology, Beijing, China) for 45 min after washing three times with TBPS. Enhanced chemiluminescent reaction (Beijing ComWin Biotechnology, Beijing, China) was used to develop the band, and ImageJ software (National Institute of Health, Maryland, USA) was used to do density analysis.

**Supplementary Table 1. Primers sequence in RT-PCR analysis**

| **Gene** | **Species** | **Sequences** |
| --- | --- | --- |
| **Adiponectin** | **mouse** | **sense: 5’-TGACGACACCAAAAGGGCTC-3’** |
|  | **mouse** | **anti-sense: 5’-ACCTGCACAAGTTCCCTTGG-3’** |
| **ADR1** | **mouse** | **sense: 5’-AGATAGGACGTGGGAGCTCAT-3’** |
|  | **mouse** | **anti-sense: 5’-ATGGCCCGTTATCAGCCAG-3’** |
| **ADR2** | **mouse** | **sense: 5’-GGAGATTTGGAGCCCAGCTT-3’** |
|  | **mouse** | **anti-sense: 5’-GGCCTTCCCACACCTTACAA-3’** |
| **AGRP** | **mouse** | **sense: 5’-GGCCTCAAGAAGACAACTGC-3’** |
|  | **mouse** | **anti-sense: 5’-GACTCGTGCAGCCTTACACA-3’** |
| **HSL** | **mouse** | **sense: 5’- TGAGATGGTAACTGTGAGCC-3’** |
|  | **mouse** | **anti-sense: 5’-ACTGAGATTGAGGTGCTGTC-3’** |
| **FOXO1** | **mouse** | **sense: 5’-TCGGCTGAATGACTGAACCT-3’** |
|  | **mouse** | **anti-sense:5’-GACCTGTACAAAGCTGGCAC-3’** |
| **PGC-1a** | **mouse** | **sense: 5’-CCGAGAATTCATGGACAAT-3’** |
|  | **mouse** | **anti-sense: 5’-GTGTGAGGAGGGTCATCGTT-3’** |
| **SIRT1** | **mouse** | **sense: 5’-CTGCCACAAGAACTAGAGGATAAGA-3’** |
|  | **mouse** | **anti-sense: 5’-TGGCAAAGGAGCAGATTAGTAGG-3’** |
| **AMPK** | **mouse** | **sense: 5’-TTGACGATGTGGCTGTGAAG-3’** |
|  | **mouse** | **anti-sense: 5’-ATAAGCCACTGCAAGCTGGT-3’** |
| **FOXO3a** | **mouse** | **sense: 5’-AGTCTCCCATGCAGACCATC-3’** |
|  | **mouse** | **anti-sense: 5’-GAGTCCGAAGTGAGCAGGTC-3’** |
| **Leptin** | **mouse** | **sense: 5’-TGGAAGCCTCACTCTACTCCA-3’** |
|  | **mouse** | **anti-sense: 5’-ACATGATTCTTGGGAGCCTGG-3’** |
| **Leptin receptor** | **mouse** | **sense: 5’-CGTTCTGCAAATCCAGGTGT-3’** |
|  | **mouse** | **anti-sense: 5’-AGTCATCGGTTGTGTTCGGT-3’** |
| **LPL** | **mouse** | **sense: 5’-ATGGAGAGCAAAGCCCTG-3’** |
|  | **mouse** | **anti-sense: 5’-TCAGCCAGCCTTCTTCAGAG-3’** |
| **NPY** | **mouse** | **sense: 5’-CCTTCCATGTGGTGATGGGA-3’** |
|  | **mouse** | **anti-sense: 5’-GCAGACTGGTTTCAGGGGAT-3’** |
| **PPAR-γ** | **mouse** | **sense: 5’-CTGTGAGACCAACAGCCTGAC-3’** |
|  | **mouse** | **anti-sense: 5’-ATGGCATCTCTGTGTCAACCAT-3’** |
| **UCP1** | **mouse** | **sense: 5’-CGACTCAGTCCAAGAGTACTTCTCTTC- 3’** |
|  | **mouse** | **anti-sense: 5’-GCCGCCTGAGATCTTGTTTC-3’** |
| **SREBP-1c** | **mouse** | **sense: 5’-GCG CTA CCG GTC TTC TAT CA- 3’** |
|  | **mouse** | **anti-sense: 5’-TGC TGC CAA AAG ACA AGG G-3’** |
| **CD36** | **mouse** | **sense: 5’-TCC TCT GAC ATT TGC AGG TCT ATC- 3’** |
|  | **mouse** | **anti-sense: 5’-GTG AAT CCA GTT ATG GGT TCC AC-3’** |
| **SCD-1** | **mouse** | **sense: 5’-CGA GGG TTG GTT GTT GAT CTG T- 3’** |
|  | **mouse** | **anti-sense: 5’-ATA GCA CTG TTG GCC CTG GA-3’** |
| **FAS** | **mouse** | **sense: 5’-GAT CCT GGA ACG AGA ACA C- 3’** |
|  | **mouse** | **anti-sense: 5’-AGA CTG TGG AAC ACG GTG GT-3’** |
| **18S** | **mouse** | **sense: 5’-TAACCCGTTGAACCCCATT-3’** |
|  | **mouse** | **anti-sense: 5’-CCATCCAATCGGTAGTAGCG-3’** |
| **GAPDH** | **mouse** | **sense: 5’-ACAGGGTGGTGGACCTCATGGT-3’** |
|  | **mouse** | **anti-sense: 5’-TGATGGTACACAAGGCAGGGCT-3’** |

**Supplementary Table 2**. The high-resolution ESI-MS chromatograms results of non-oligosaccharide fraction in PSE.

| Number^a^ | Molecular weight | Possible compounds |
| --- | --- | --- |
| 1 | 578; 866; 1152 | B-type dimers; B-type trimers; B-type tetramers |
| 2 | 578; 864; 1152 | B-type dimers; A-type trimers; B-type tetramers |
| 3 | 578; 864; 1152 | B-type dimers; A-type trimers; B-type tetramers |
| 4 | 864; 1150 | A-type trimers; A-type tetramers |
| 5 | 578; 864; 1152 | B-type dimers; A-type trimers; B-type tetramers |
| 6 | 864; 1152 | A-type trimers; B-type tetramers |
| 7 | 578; 864; 1150 | B-type dimers; A-type trimers; A-type tetramers |
| 8 | 576; 864; 1150 | A-type dimers; A-type trimers; A-type tetramers |
| 9 | 576; 864; 1152 | A-type dimers; A-type trimers; B-type tetramers |
| 10 | 576; 864; 1150; 1152 | A-type dimers; A-type trimers; A-type tetramers; B-type tetramers |

^a^ Numbering of peaks in Supplementary Fig. 10.


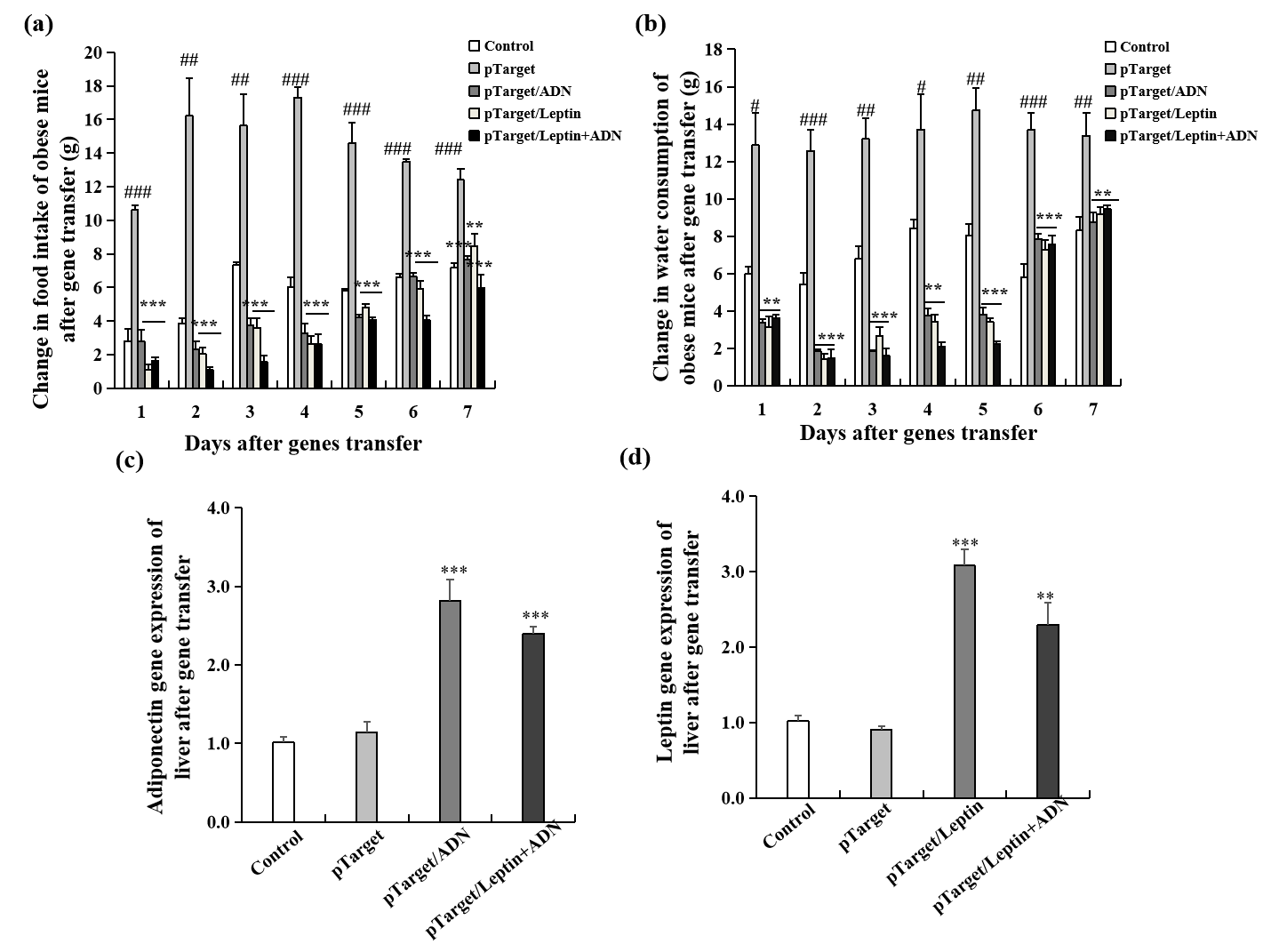


**Supplementary Figure 1.** Effects of leptin, adiponectin and leptin plus adiponectin gene transfer on high fat-induced obese mice. The changes of food intake (a) and water consumption (b) of obese after gene transfer. The changes of adiponectin (c) and leptin genes (d) expression in liver of mice at end of experiment after gene transfer. Animal numbers of each group are eight. ^#, ##, ###^ represent significant difference between control group and pTarget vector group obese mice group at p< 0.05, p< 0.01 and p< 0.001. ^**, ***^represent significant difference between pTarget vector group and gene transfer group at p< 0.01 and p< 0.001.

**
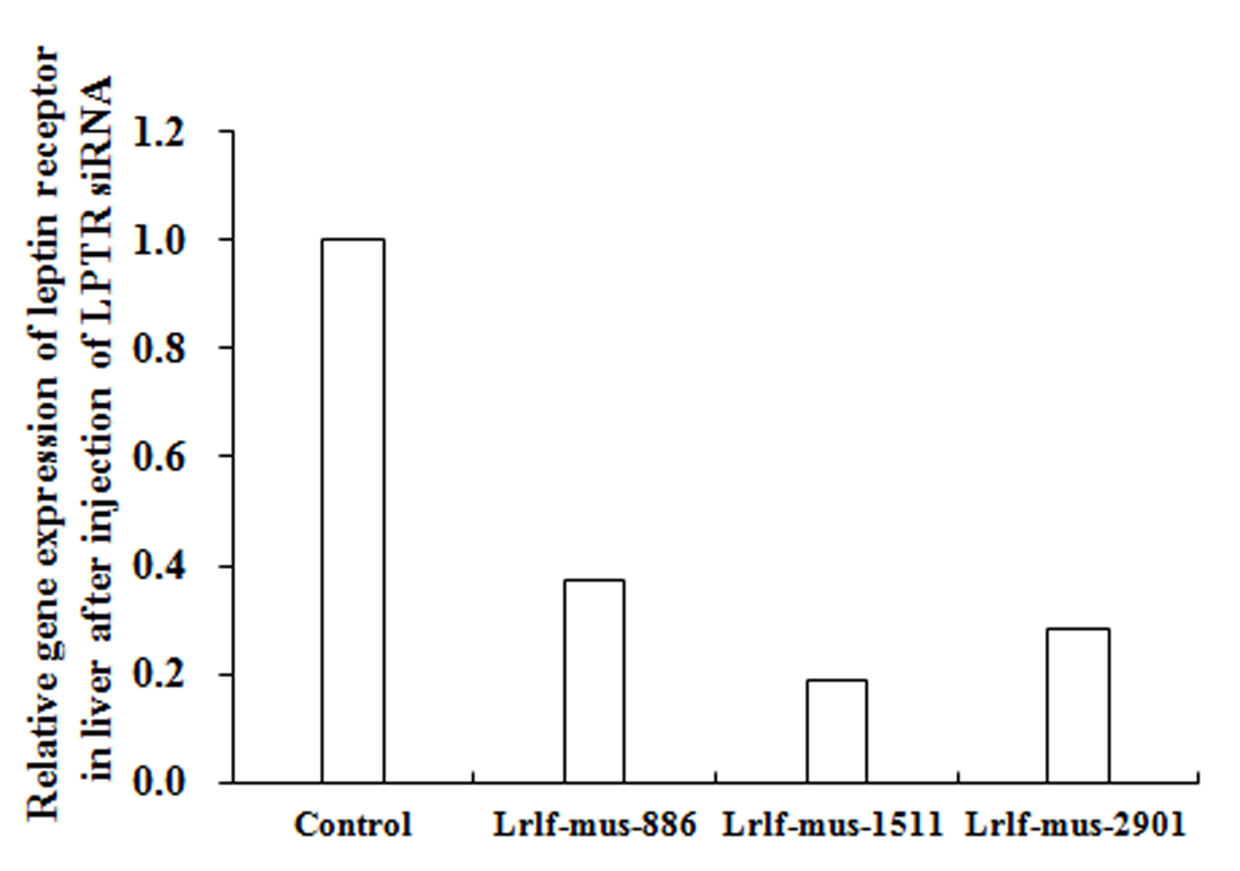
**

**Supplementary Figure 2.** Effects of different point siRNA of leptin receptor on gene expression of leptin receptor in liver of mouse *in* *vivo.* The gene expression of leptin receptor in liver after administrating siRNA of leptin receptor at a dose of 16 μg with hydrodynamics based-gene delivery.

**
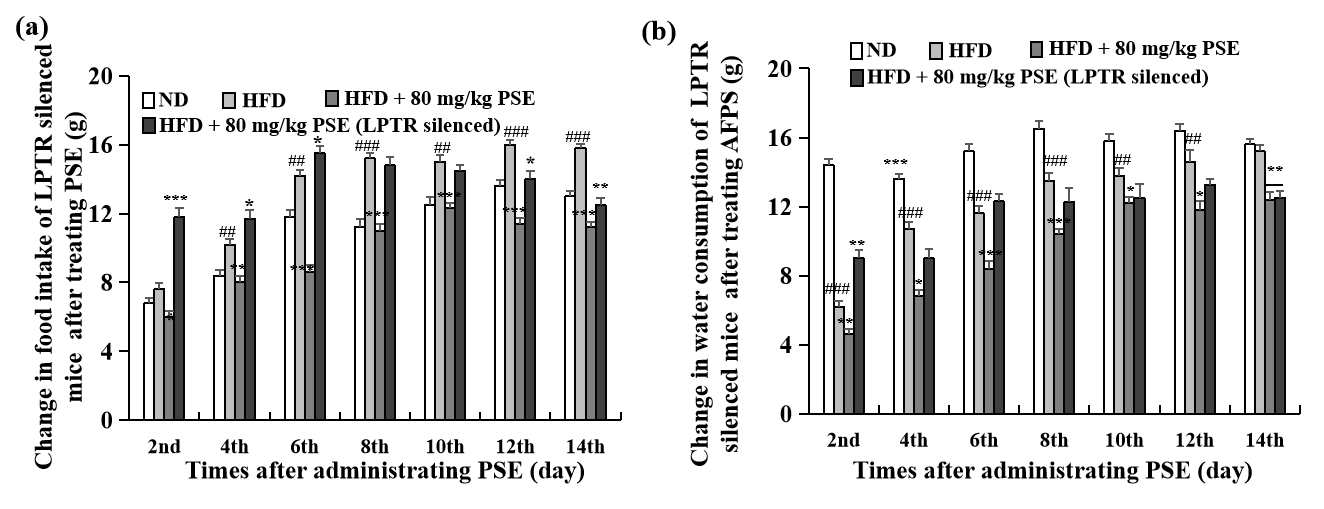
**

**Supplementary Figure 3.** Effects of siRNA leptin receptor and PSE on the food intake and water consumption of high fat-induced obese mice. The changes of food intake (a) and water consumption (b) of obese mice after administrating siRNA and PSE. Animal numbers of each group are eight. ^##, ###^ represent significant difference between control group and HFD group at p< 0.01 and p< 0.001. ^*, **, ***^represent significant difference between HFD group and siRNA leptin receptor and PSE treated groups at p< 0.05, p< 0.01 and p< 0.001.


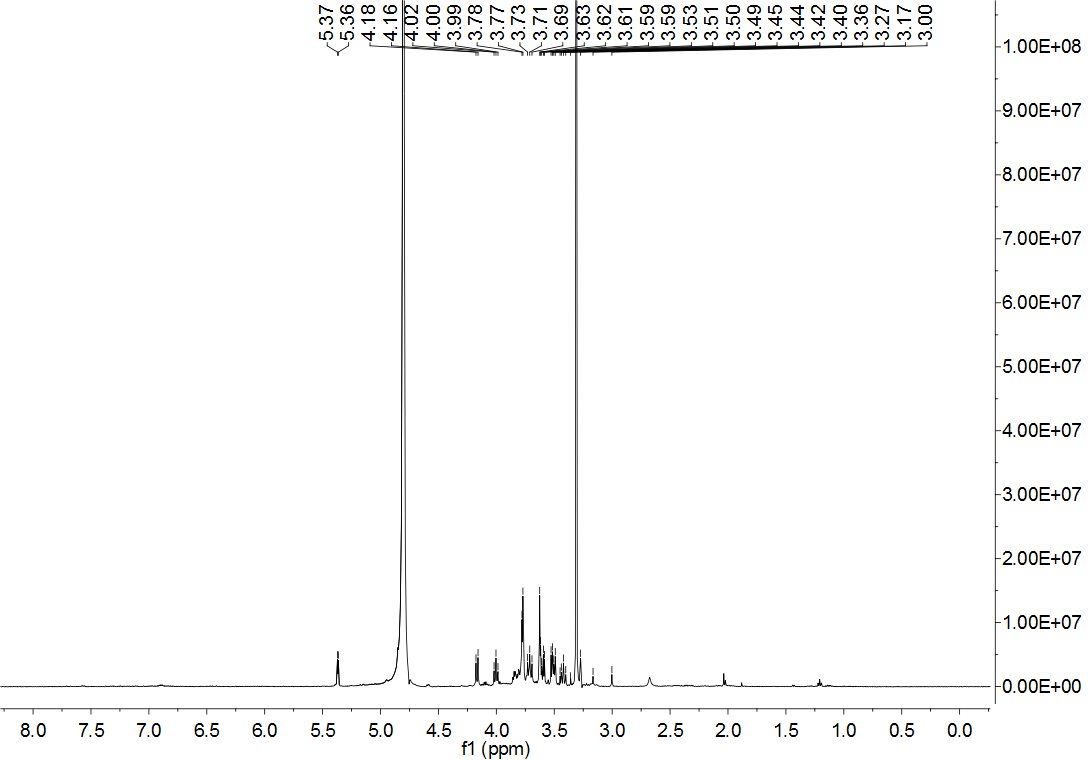


**Supplementary Figure 4**. ^1^H-NMR spectrum of oligosaccharide fraction of PSE in CD_3_OD.

**
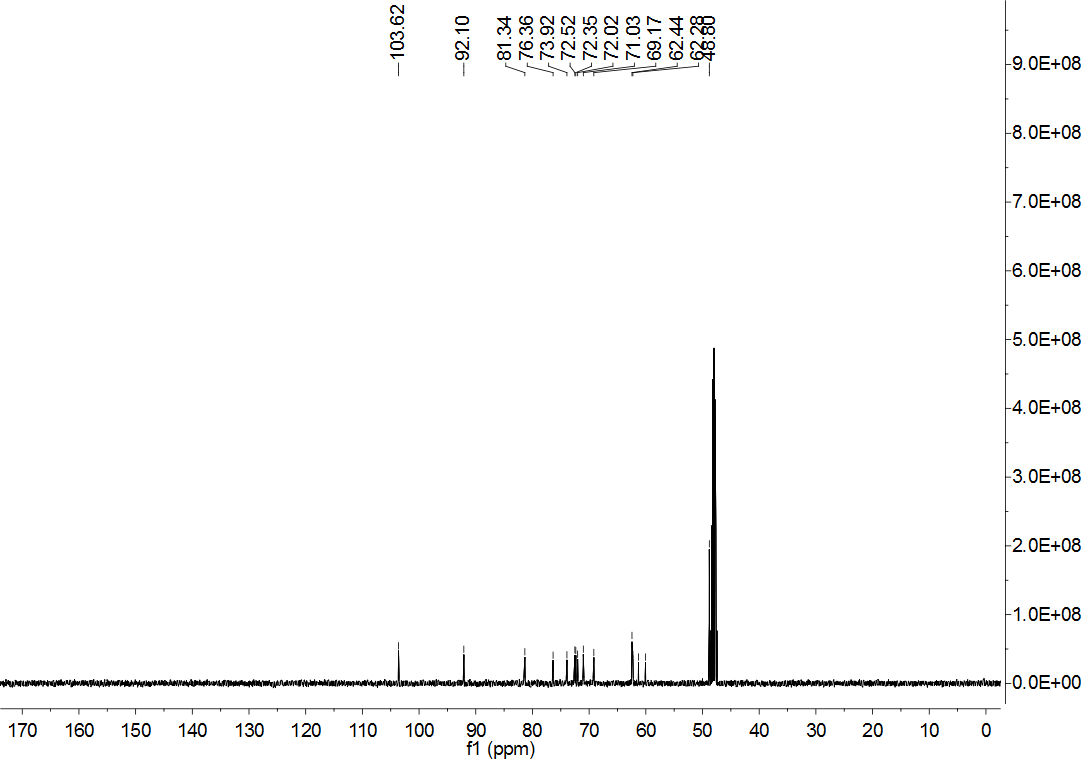
**

**Supplementary Figure 5**. ^13^C-NMR spectrum of oligosaccharide fraction of PSE in CD_3_OD.

**
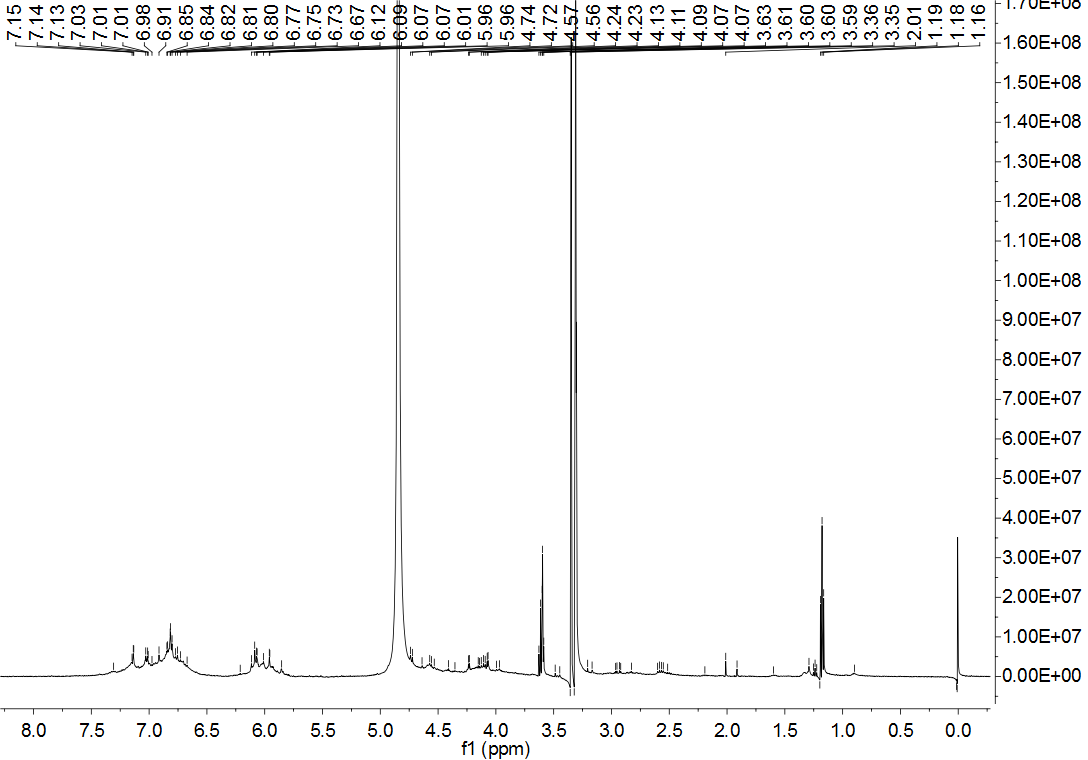
**

**Supplementary Figure 6**. ^1^H-NMR spectrum of non-oligosaccharide fraction of PSE in CD_3_OD.


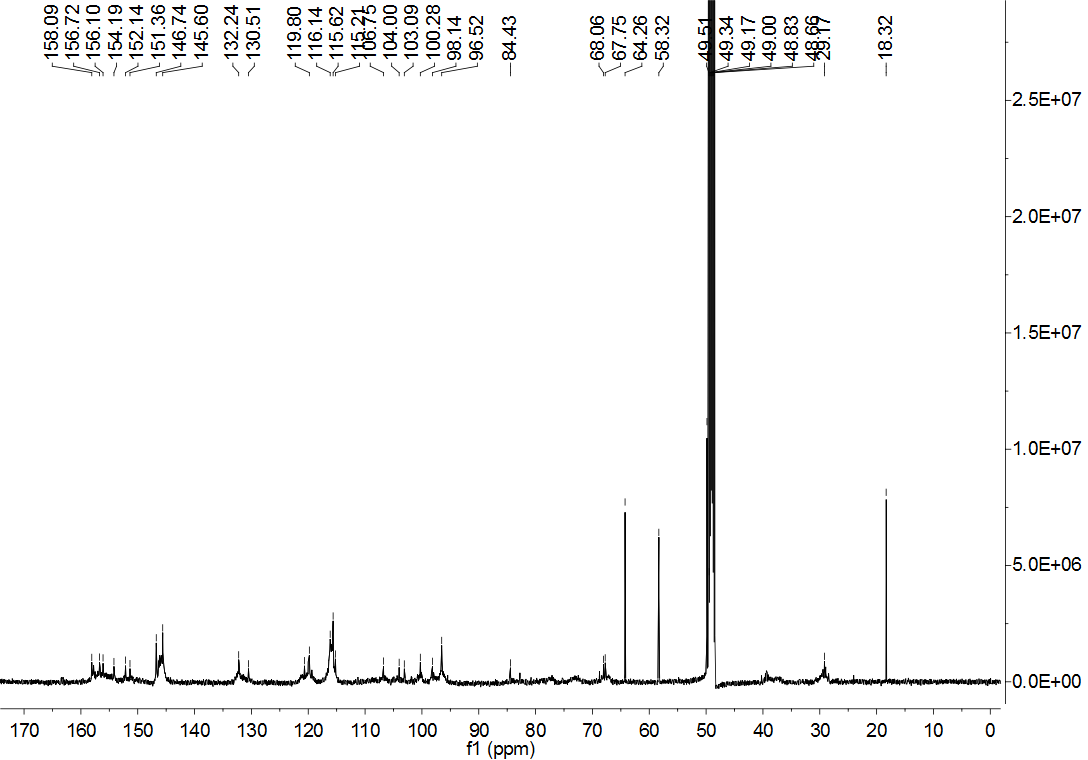


**Supplementary Figure 7**. ^13^C-NMR spectrum of non-oligosaccharide fraction of PSE in CD_3_OD.


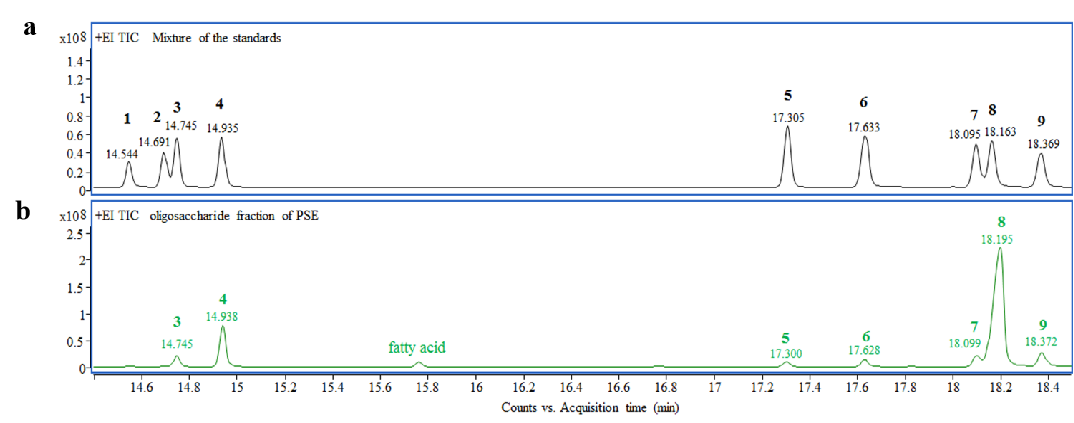


**Supplementary Figure 8**. GC/MS chromatograms of the monosaccharides in the standard mixture (**a**), oligosaccharide fraction of PSE (**b**). Peak identity: **1**, rhamnose; **2**, fucose; **3**, arabinose; **4**, xylose; **5**, D-(+)-chiro-inositol; **6**, myo-inositol; **7**, mannose; **8**, glucose; **9**, galactose.


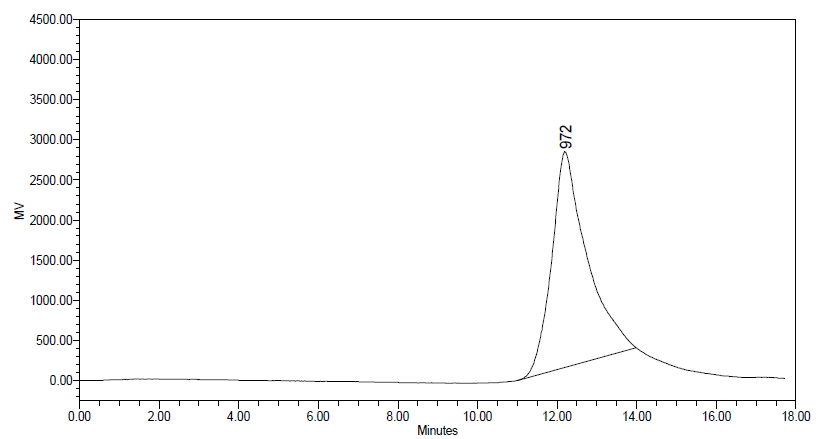


**Supplementary Figure 9**. The average molecular weight (Mw) of oligosaccharide fraction of PSE.


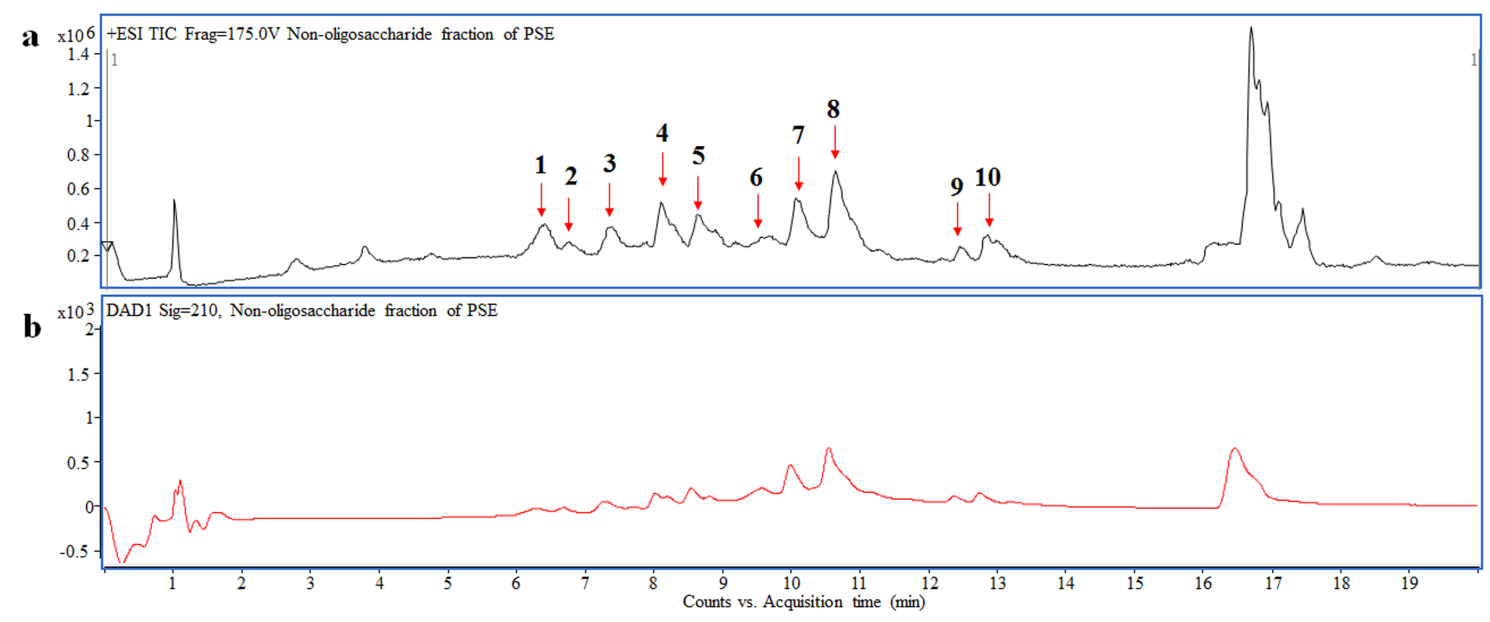


**Supplementary Figure 10**. High-resolution ESI-MS chromatograms of non-oligosaccharide fraction of PSE in ESI spectrum (a) and DAD spectrum (b). Peaks with number were displayed in supplementary Table 2.

**
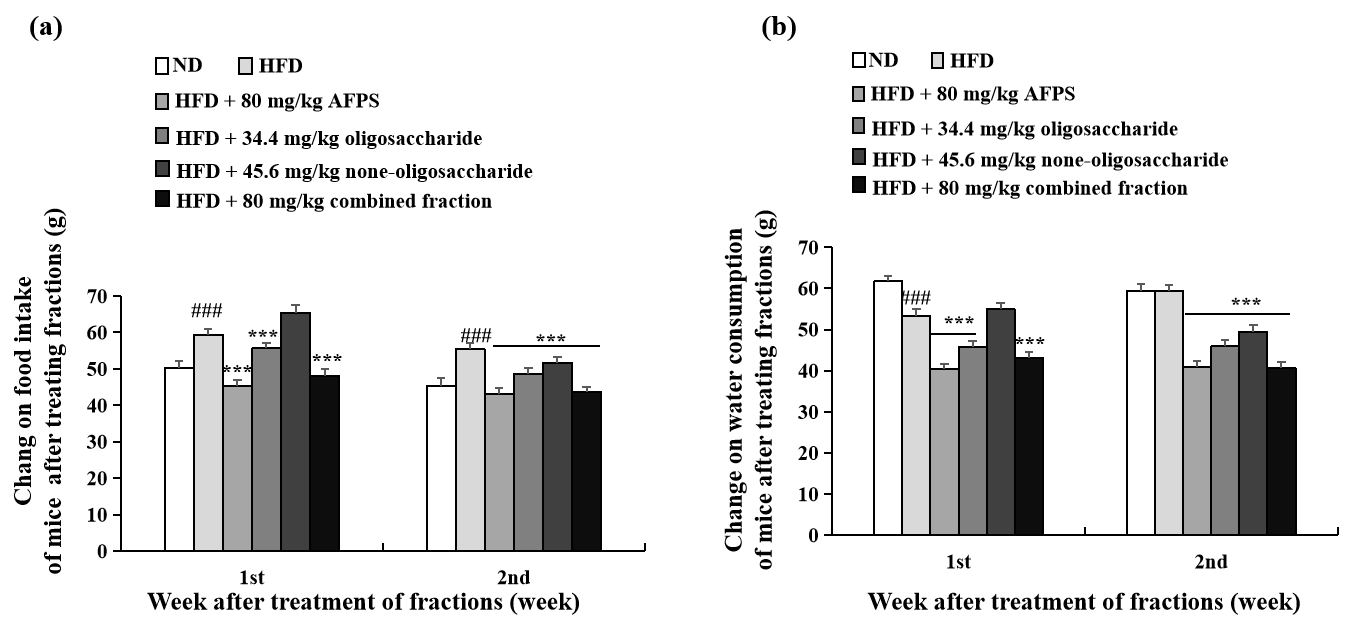
**

**Supplementary Figure 11.** Effects of oligosaccharide and non-oligosaccharide fractions of PSE on the food intake and water consumption of high fat-induced obese mice. The changes of food intake (a) and water consumption (b) of obese mice after administrating oligosaccharide and non- oligosaccharide fractions of PSE and PSE, respectively. Animal numbers of each group are eight. ^###^ represents significant difference between control and HFD groups at p< 0.001. ^***^represents significant difference between HFD group and treatment groups at p< 0.001.


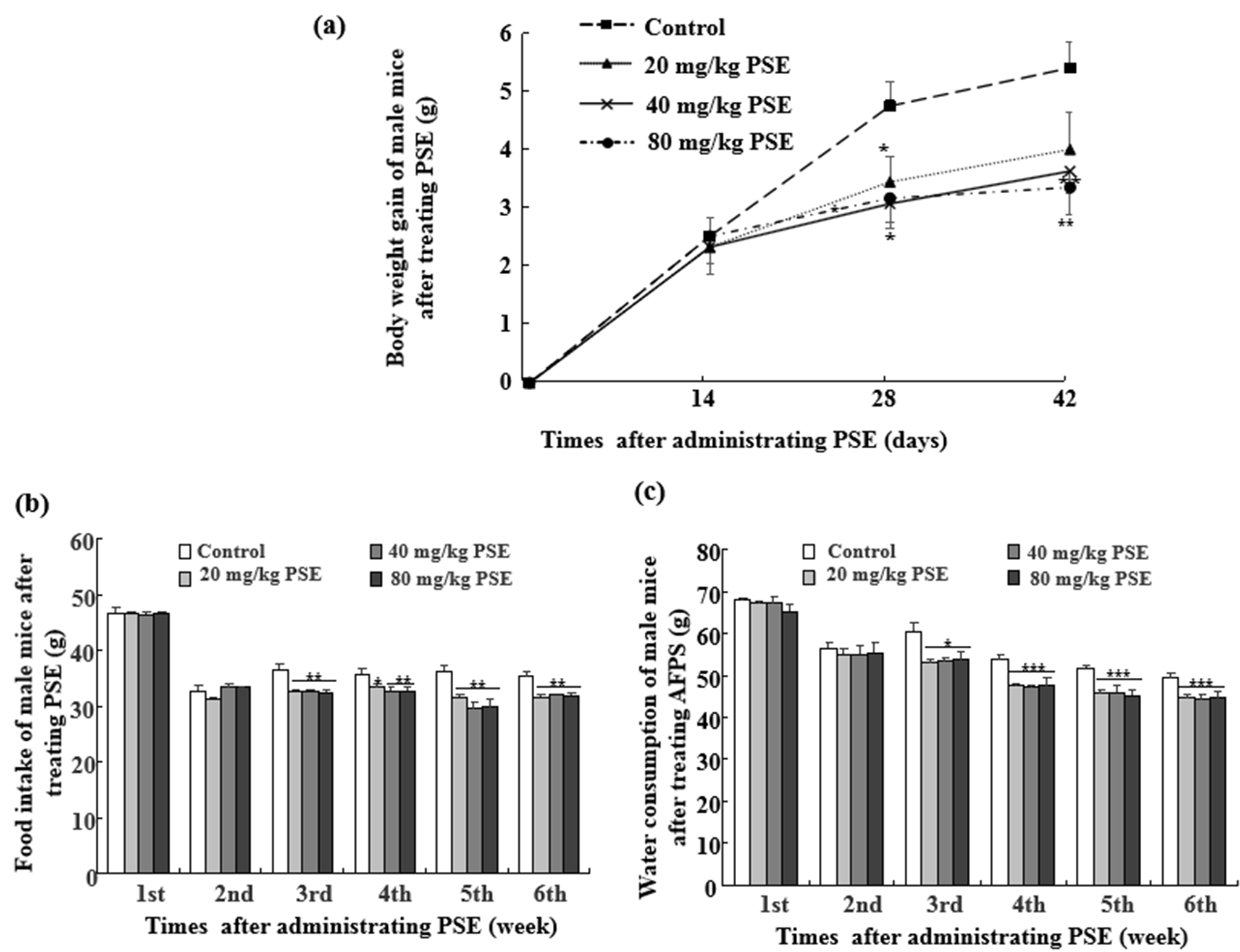


**Supplementary Figure 12.** Anti-obesity effects of PSE for normal male mice. The change of body weight gain (a), food intake (b), and water consumption (c) of normal male mice after administrating AFPS at 20, 40, and 80 mg/kg for six weeks. Each point in figure represents the Mean ± SEM at corresponding time points. Animal numbers of each group are ten, and the experiment is repeated twice. *, ** and *** indicate significant difference compared with control group at p< 0.05, p< 0.01 and p< 0.001.


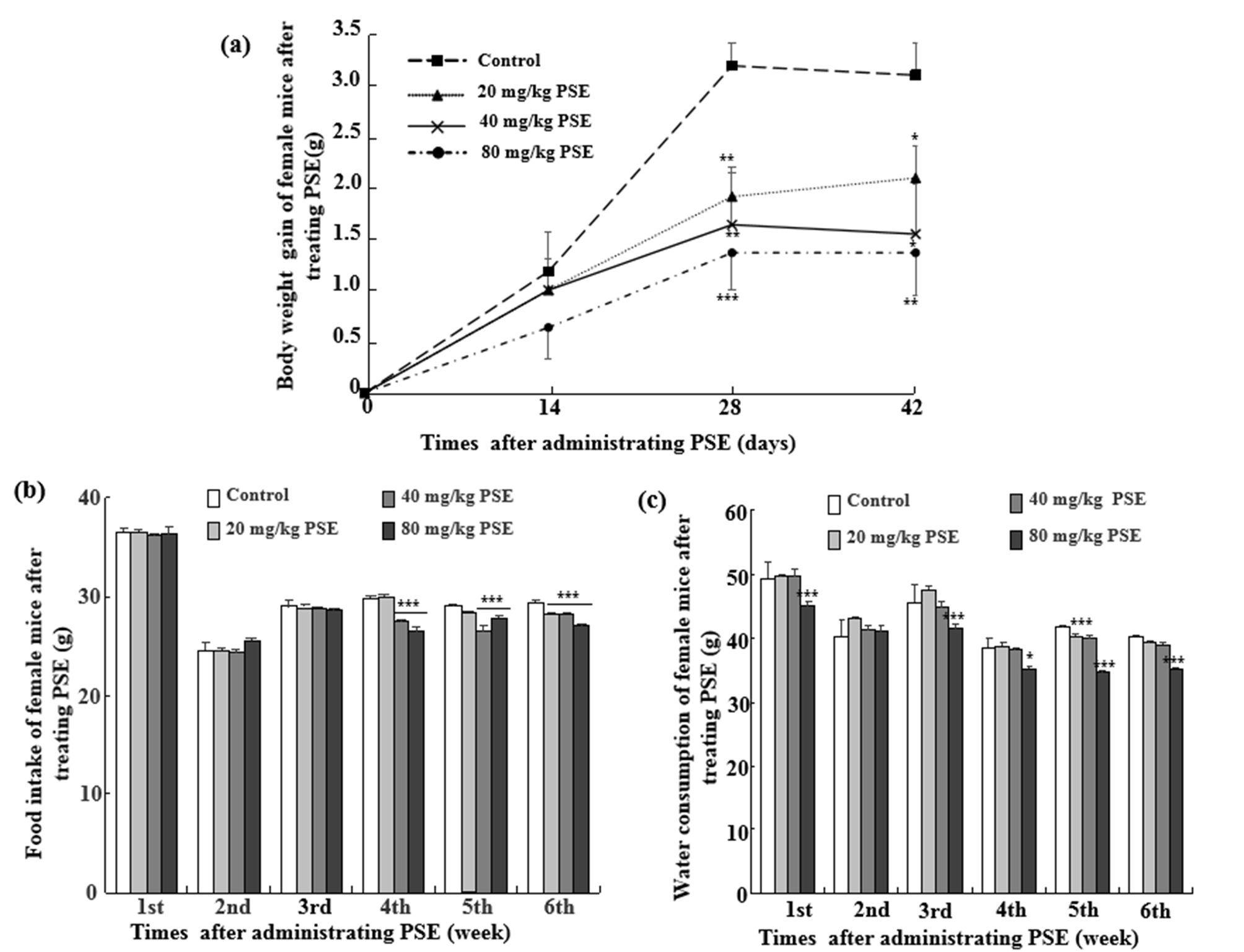


**Supplementary Figure 13.** Anti-obesity effects of PSE for normal female mice. The change of body weight (a), food intake (b), and water consumption (c) of normal female mice after administrating PSE at 20, 40, and 80 mg/kg for six weeks. Each point in figure represents the Mean ± SEM at corresponding time points. Animal numbers of each group are ten, and the experiment is repeated twice. ^*, **^ and ^***^ indicate significant difference comparison with HFD group at p< 0.05, p< 0.01 and p< 0.001.
